# Supplementary material for: Shear localisation controls the dynamics of earthquakes
Source: Nat Commun. 2025 Jan 16;16:711. doi: 10.1038/s41467-024-55363-y (PMC11739507; doi:10.1038/s41467-024-55363-y)
Supplement: Supplementary file 1 — Supplementary Information [file 41467_2024_55363_MOESM1_ESM.pdf]

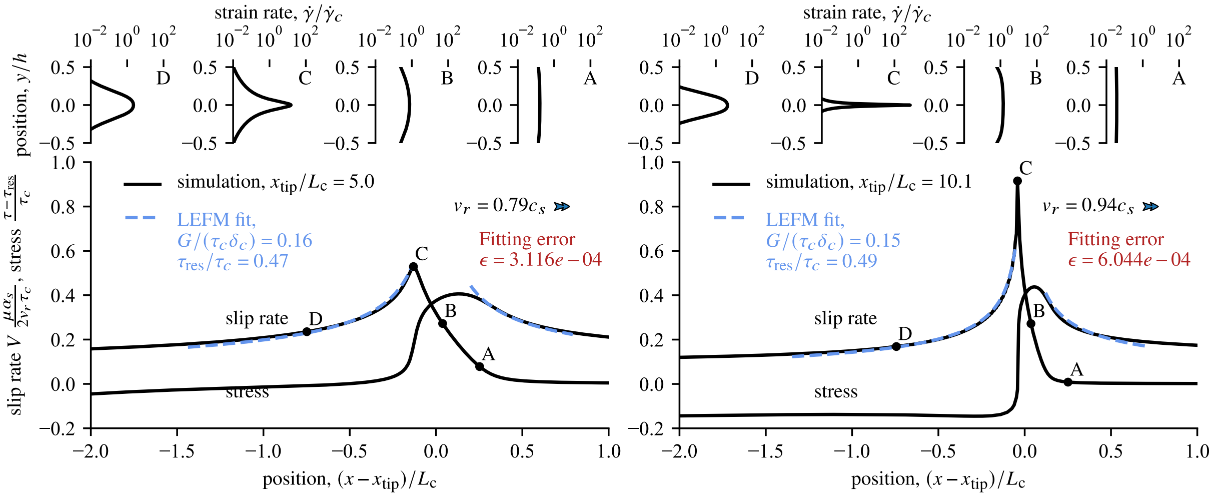

Supplementary Figure 1: Two additional snapshots of the same seismic rupture that shown in Figure 4 but observed at different locations along the interface. The error of the fitting procedure is also given to quantify the agreement with dynamic fracture predictions.

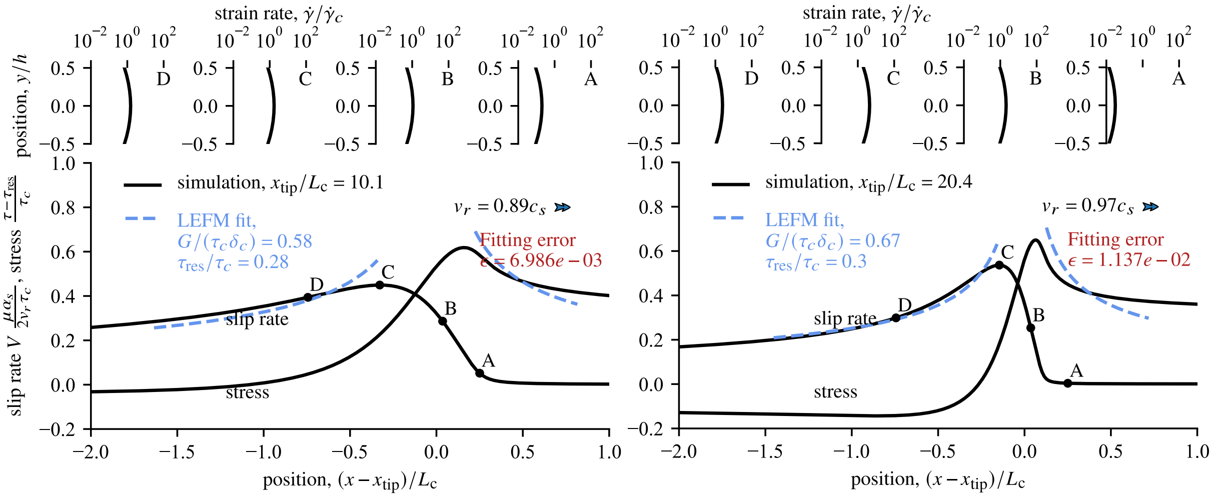

Supplementary Figure 2: Two snapshots of the seismic rupture shown by the brown data in Figure 5 and simulated with a model that prevents strain localisation. The organisation of the figure is similar to the one of Figure 4 and 1.
